# Estimating the Inertia Tensor Components of an Asymmetrical Spacecraft When Removing It from the Operational Orbit at the End of Its Active Life

**DOI:** 10.3390/s23239615

**Published:** 2023-12-04

**Authors:** A. V. Sedelnikov, D. I. Orlov, M. E. Bratkova, E. S. Khnyryova

**Affiliations:** 1 Department of Theoretical Mechanics, Samara National Research University, Samara 443086, Russia; bratkova02@mail.ru; 2 Department of Space Engineering, Samara National Research University, Samara 443086, Russia; grand_99v@mail.ru (D.I.O.); khnyryova@gmail.com (E.S.K.)

**Keywords:** estimating the inertia tensor, space debris, magnetometer, space tug

## Abstract

The paper presents a method for estimating the inertia tensor components of a spacecraft that has expired its active life using measurement data of the Earth’s magnetic field induction vector components. The implementation of this estimation method is supposed to be carried out when cleaning up space debris in the form of a clapped-out spacecraft with the help of a space tug. It is assumed that a three-component magnetometer and a transmitting device are attached on space debris. The parameters for the rotational motion of space debris are estimated using this measuring system. Then, the known controlled action from the space tug is transferred to the space debris. Next, measurements for the rotational motion parameters are carried out once again. Based on the available measurement data and parameters of the controlled action, the space debris inertia tensor components are estimated. It is assumed that the measurements of the Earth’s magnetic field induction vector components are made in a coordinate system whose axes are parallel to the corresponding axes of the main body axis system. Such an estimation makes it possible to effectively solve the problem of cleaning up space debris by calculating the costs of the space tug working body and the parameters of the space debris removal orbit. Examples of numerical simulation using the measurement data of the Earth’s magnetic field induction vector components on the Aist-2D small spacecraft are given. Thus, the purpose of this work is to evaluate the components of the space debris inertia tensor through measurements of the Earth’s magnetic field taken using magnetometer sensors. The results of the work can be used in the development and implementation of missions to clean up space debris in the form of clapped-out spacecraft.

## 1. Introduction

Nowadays, various projects are being developed to clean up space debris from near-Earth space. This issue was first raised at UN meetings in the early 1980s. Even then, it became clear that the active use of near-Earth space would create the problem of its cleaning from space debris of terrestrial origin [1,2]. Space debris poses a serious threat to the safe operation of unmanned and manned spacecraft in near-Earth orbits. Due to the threat of collision with space debris, maneuvers have become common practice in the operation of modern spacecraft [3,4]. All experts note that the number of launches of small spacecraft will increase significantly in the future [5].

Therefore, in the opinion of many authors, nowadays, it is necessary to design spacecraft with specific systems for its removal from the orbit at the end of its active life [6].

Various concepts have been developed to remove space debris from near-Earth orbits. The authors of [7] believe that the standard propulsion system of the spacecraft and the remnants of the working fluid can be used for removal. In this case, it is not necessary to design a specific system that removes the spacecraft at the end of its active life. However, this method can be used for the oriented flight of the spacecraft with a full-fledged motion control system. The use of executive bodies that do not require the expenditure of working fluid makes this method inefficient.

The work [8] considers a drag augmentation system (DAS), which is a space sail [9] that unfolds at the end of the spacecraft’s active life. This sail contributes to removing the spacecraft from the orbit due to the aerodynamic drag increase. This method involves the development of a specific system for transporting and unfolding the sail and is applicable mainly in low near-Earth orbits. For high orbits, the spacecraft deorbit time can be significant. Modern materials of such a sail have high stress–strain properties and a low specific gravity. Therefore, the increase in the mass parameters of a small spacecraft when using such a system will be insignificant.

**Figure 1:**
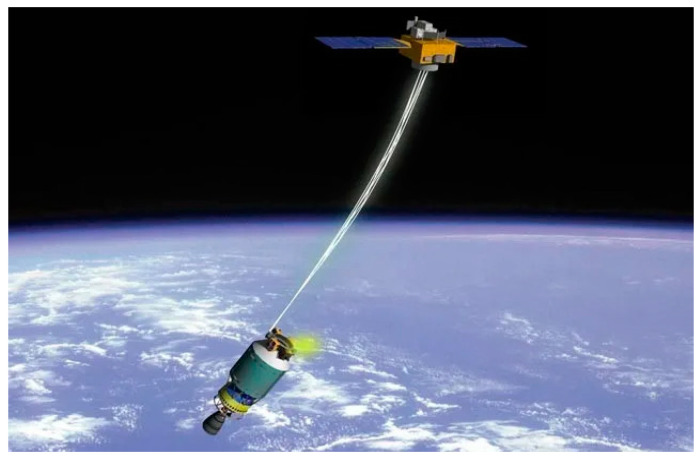
A method for removing space debris using a space tug and a tether system.

The review [10] presents a comparative analysis of four different methods to remove spacecraft from low near-Earth orbits at the end of their active life. Two active devices (classical rocket and electric motors) and two passive technologies (drag augmentation devices and cables of electrodynamic tether systems [10]) are considered. The authors of [10] believe that, with other factors being equal, for an initial height of 850 km, cables are approximately one and two orders of magnitude lighter than active devices and drag augmentation devices, respectively. In this case, special attention is paid to electrodynamic tether systems, according to the results of the FP7/Space BETs project [10]. The superiority of ribbon cables over round and wire cables in terms of deorbit efficiency is substantiated, as well as the importance of the optimal choice for the length, width, and thickness of a ribbon cable depending on the spacecraft mass and its initial orbit [10]. Figure 1 shows a scheme of transporting space debris by a cable using a space tug [11].

The prospect of using tether systems is noted by many researchers, for example, the authors of [12,13,14,15]. It is possible to design and install systems for deorbiting the spacecraft when creating new space technology, but the task of cleaning up existing space debris leaves significantly fewer options for its solution. Therefore, one of the promising options for such cleaning is the use of a space tug in combination with a tether system for transporting space debris.

At the same time, methods of non-contact debris removal are actively developed, for example, using a laser system [16]. The authors of [16] propose to create a space laser facility to protect orbital stations from space debris. Based on the results of numerical simulation, a design for a space-based laser system was proposed in [16]. The developed laser system can effectively deal with space debris ranging in size from 1 to 10 cm. However, this method is more suitable for the protection of operating space objects than for cleaning up space debris.

The work [17] contains a detailed review and comparison of existing technical solutions and approaches to space debris removal. Contactless transport systems are considered as a promising direction in creating safe and reliable space debris removal systems. The use of an ion beam is proposed as one of the active influences on space debris [17,18]. The work [18] presents a scheme of the ion beam’s impact on space debris and analyzes the parameters of the impact that is necessary to solve the problem of its removal successfully [18]. In [19], a multipath scheme was proposed and control laws for impulse motors were developed.

For effective contact (through tether systems) and the contactless (via ion beams) cleaning of space debris in the form of spacecraft that have exhausted their active life, it is necessary to know the inertial mass parameters of these spacecraft. Therefore, the problem of estimating the inertial mass parameters of space debris, as well as the parameters of its rotational motion in absolute space and relative to the space tug, arises. This problem was considered not only in the context of the space debris problem in [11,20,21,22]. In [20], the difficulties of estimating the inertia tensor of a captured object are noted in the case when the connection between the space tug and the debris is not rigid, for example, when using a tether system.

In [20], the components of the space debris inertia tensor are estimated using various Kalman filters by measuring the rotation velocity of space debris. The cases of a cable stretched all the time and a cable subject to frequent weakening are considered. A good estimation quality is shown if the cable tension and the cable attachment point are known [20]. However, in some cases, the authors of [20] note a large dispersion of the obtained estimations.

In [21], the traditional method was employed to achieve an accurate estimation of the inertial mass parameters in a system analysis designed for the errors which influence the parameter measurements of the space debris rotational motion. To improve the estimation accuracy, the authors of [21] proposed a modification for the estimation equations by including the data of the space tug contact force impact on space debris.

In [22], it was proposed to use a nanosatellite as a data measurement system for estimating the parameters of the space debris rotational motion. This satellite must dock with space debris and move with it as a single body. However, docking issues are not discussed.

In general formulation, solving the problem of estimating the inertia tensor components of arbitrary-shaped space debris moving arbitrarily in outer space is quite complicated. The possibility of attaching several measuring instruments on different parts of space debris, and the possibility of monitoring the relative position of these instruments, while taking into account errors, will expand the range of the proposed method application for estimating the inertia tensor components. However, technically, it is not easy to solve this problem.

This work makes the following contribution:

(1) A method for estimating the inertia tensor components of space debris and the parameters of its rotational motion by attaching elements of the data-measuring system on a space debris object is proposed;
(2) A simulation is carried out for a particular case of attaching measuring instruments on a space debris object;
(3) The results of numerical simulation for a particular case with an estimation of inertia tensor components for the Aist-2D small spacecraft are presented;
(4) An analysis of the obtained results was carried out and recommendations for its use were given.

## 2. Problem Formulation

**Figure 2:**
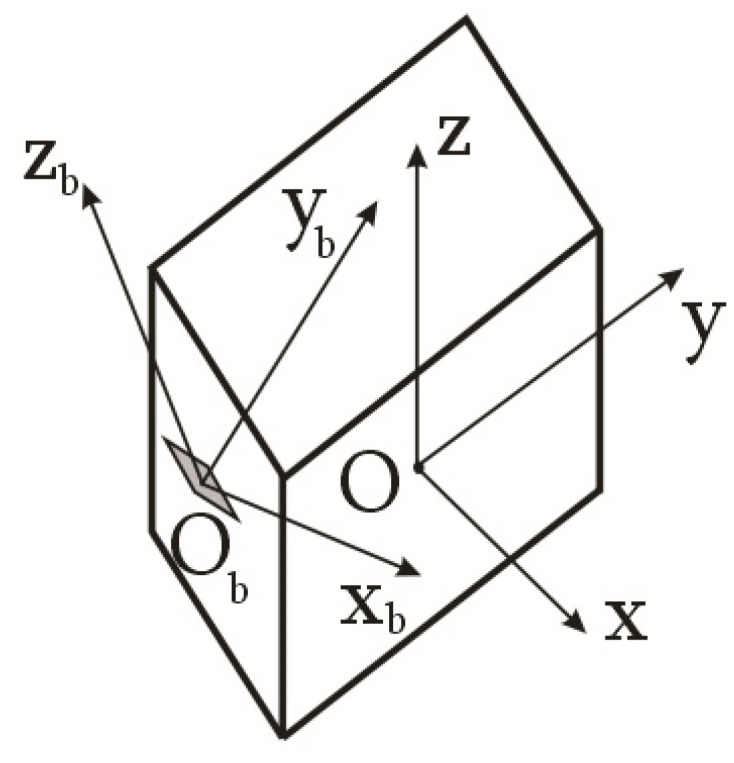
Scheme of attaching the magnetometer on the space debris object in an arbitrary case: Oxyz is the main body axis system of the space debris object; O_b_x_b_y_b_z_b_ is the structural coordinate system of the magnetometer.

Let us consider the problem of estimating the inertia tensor components for a space debris object in the general formulation within the framework of the proposed approach of attaching the measuring equipment—a magnetometer—on it. Let us assume that a three-component magnetometer with a data-transmitting device has been attached to the space debris object. In this case, using the measurements of the Earth’s magnetic field induction vector, it is possible to estimate the components of the angular velocity vector of space debris in the magnetometer’s structural coordinate system (Figure 2).

**Figure 3:**
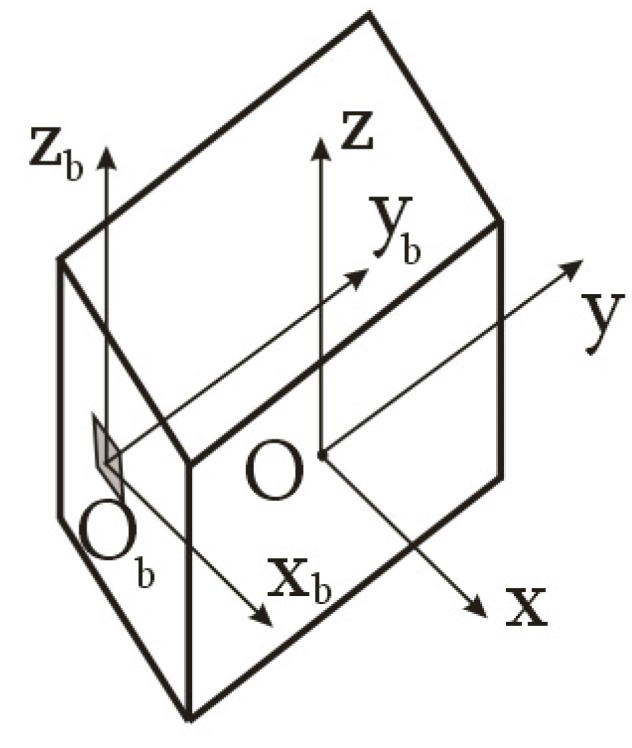
Scheme of attaching the magnetometer on the space debris object in the special case.

To obtain a correct estimation for the angular velocity vector, it is proposed in [23] to use the derivative of the Earth’s magnetic field induction vector components:

(1) $${\vec{\omega }}_{k}=\frac{{\dot{\vec{B}}}_{k}\times {\dot{\vec{B}}}_{k-1}}{\Delta t_{k}{\left|{\dot{\vec{B}}}_{k}\right|}^{2}},$$

where ${\dot{\vec{B}}}_{k}\left({\dot{B}}_{xk},\;{\dot{B}}_{yk},\;{\dot{B}}_{zk}\right)$ and ${\dot{\vec{B}}}_{k-1}\left({\dot{B}}_{xk-1},\;{\dot{B}}_{yk-1},\;{\dot{B}}_{zk-1}\right)$ are the derivatives of the Earth’s magnetic field induction vector components and its components in the magnetometer’s structural coordinate system (Figure 3) for the *k*-th and *k* − 1-th measurements, respectively; $\Delta t_{k}=t_{k}-t_{k-1}$ is the time interval between *k*-th and *k* − 1-st measurements.

Let us represent the vector Equation (1) in the axes of the magnetometer’s structural coordinate system (Figure 3):

(2) $$\left\{\begin{array}{l} \omega_{xk}=\frac{{\dot{B}}_{yk}{\dot{B}}_{zk-1}-{\dot{B}}_{zk}{\dot{B}}_{yk-1}}{\Delta t_{k}\left({\dot{B}}_{xk}^{2}+{\dot{B}}_{yk}^{2}+{\dot{B}}_{zk}^{2}\right)};\\ \omega_{yk}=\frac{{\dot{B}}_{zk}{\dot{B}}_{xk-1}-{\dot{B}}_{xk}{\dot{B}}_{zk-1}}{\Delta t_{k}\left({\dot{B}}_{xk}^{2}+{\dot{B}}_{yk}^{2}+{\dot{B}}_{zk}^{2}\right)};\\ \omega_{zk}=\frac{{\dot{B}}_{xk}{\dot{B}}_{yk-1}-{\dot{B}}_{yk}{\dot{B}}_{xk-1}}{\Delta t_{k}\left({\dot{B}}_{xk}^{2}+{\dot{B}}_{yk}^{2}+{\dot{B}}_{zk}^{2}\right)}. \end{array}\right.$$

Then, with an arbitrary location of the axes of the magnetometer’s structural coordinate system relative to the main body axis system of the space debris object, the Euler dynamic equations in the magnetometer’s structural coordinate system will have the form [24]:

(3) $$\left\{\begin{array}{l} I_{xx}{\dot{\omega }}_{xk}-I_{xy}{\dot{\omega }}_{yk}-I_{xz}{\dot{\omega }}_{zk}+\omega_{yk}\left(I_{zz}\omega_{zk}-I_{xz}\omega_{xk}-I_{yz}\omega_{yk}\right)-\omega_{zk}\left(I_{yy}\omega_{yk}-I_{xy}\omega_{xk}-I_{yz}\omega_{zk}\right)=M_{x}\\ I_{yy}{\dot{\omega }}_{yk}-I_{xy}{\dot{\omega }}_{xk}-I_{yz}{\dot{\omega }}_{zk}+\omega_{zk}\left(I_{xx}\omega_{xk}-I_{xy}\omega_{yk}-I_{xz}\omega_{zk}\right)-\omega_{xk}\left(I_{zz}\omega_{zk}-I_{xz}\omega_{xk}-I_{yz}\omega_{yk}\right)=M_{y}\\ I_{zz}{\dot{\omega }}_{zk}-I_{xz}{\dot{\omega }}_{xk}-I_{yz}{\dot{\omega }}_{yk}+\omega_{xk}\left(I_{yy}\omega_{yk}-I_{xy}\omega_{xk}-I_{yz}\omega_{zk}\right)-\omega_{yk}\left(I_{xx}\omega_{xk}-I_{xy}\omega_{yk}-I_{xz}\omega_{zk}\right)=M_{z} \end{array}\right.,$$

where $\vec{M}\left(M_{x},\;M_{y},\;M_{z}\right)$ is the main vector of external moments acting on the space debris object; $\hat{I}=\left[\begin{array}{l} I_{xx}\;I_{xy}\;I_{xz}\\ I_{xy}\;I_{yy}\;I_{yz}\\ I_{xz}\;I_{yz}\;I_{zz} \end{array}\right]$ is the symmetrical inertia tensor in the magnetometer’s structural coordinate system; ${\dot{\vec{\omega }}}_{k}\left({\dot{\omega }}_{xk},\;{\dot{\omega }}_{yk},\;{\dot{\omega }}_{z}\right)$ is the derivative of the angular velocity vector of the space debris object and its components in the magnetometer’s structural coordinate system (Figure 2).

Further, the known perturbing effect is transferred to the space debris object. The above equations are then also used to estimate the rotational motion parameters.

In the general formulation, the problem of estimating inertia tensor components of the space debris object using measurements of a single magnetometer cannot be solved without additional data.

Therefore, let us consider a special case, whereby the origin of the magnetometer’s structural coordinate system is located on one of the axes of the main body axis system, and the *O_b_x_b_y_b_z_b_* axes of the structural coordinate system and *Oxyz* axes of the main body axis systems are parallel (Figure 3).

In this case, taking into account the introduced simplified assumption, Equation (3) is transformed to the form [24]:

(4) $$\left\{\begin{array}{l} I_{xx}{\dot{\omega }}_{xk}+\omega_{yk}\omega_{zk}\left(I_{zz}-I_{yy}\right)=M_{x}\\ I_{yy}{\dot{\omega }}_{yk}+\omega_{xk}\omega_{zk}\left(I_{xx}-I_{zz}\right)=M_{y}\\ I_{zz}{\dot{\omega }}_{zk}+\omega_{xk}\omega_{yk}\left(I_{yy}-I_{xx}\right)=M_{z} \end{array}\right..$$

Let us rewrite Equation (4) with respect to the diagonal inertia moments in the structural coordinate system:

(5) $$\left\{\begin{array}{l} {\dot{\omega }}_{xk}I_{xx}-I_{yy}\omega_{yk}\omega_{zk}+\omega_{yk}\omega_{zk}I_{zz}=M_{x}\\ {\dot{\omega }}_{yk}I_{yy}+\omega_{xk}\omega_{zk}I_{xx}-\omega_{xk}\omega_{zk}I_{zz}=M_{y}\\ {\dot{\omega }}_{zk}I_{zz}-\omega_{xk}\omega_{yk}I_{xx}+\omega_{xk}\omega_{yk}I_{yy}=M_{z} \end{array}\right..$$

Let us assume that the quantity of the controlled action is significant enough to neglect the external disturbing action. Then, the right parts of Equation (5) will represent the moment from the controlled action in the magnetometer’s structural coordinate system (Figure 3):

(6) $$\vec{M}=\vec{r}\times {\vec{F}}_{cont},$$

where $\vec{r}$ is the radius vector of the controlled action application point relative to the origin of the magnetometer’s structural coordinate system; ${\vec{F}}_{cont}$ is the vector of the controlled action.

Let us express the diagonal components of the inertia tensor from system (5):

(7) $$\left\{\begin{array}{l} I_{zz}=\frac{M_{z}{\dot{\omega }}_{xk}+M_{x}\omega_{xk}\omega_{yk}-\left(M_{y}{\dot{\omega }}_{xk}-M_{x}\omega_{xk}\omega_{zk}\right)\frac{\omega_{xk}\omega_{yk}^{2}\omega_{zk}-\omega_{xk}\omega_{yk}{\dot{\omega }}_{xk}}{{\dot{\omega }}_{xk}{\dot{\omega }}_{yk}+\omega_{xk}\omega_{yk}\omega_{zk}^{2}}}{{\dot{\omega }}_{xk}{\dot{\omega }}_{zk}+\omega_{xk}\omega_{yk}^{2}\omega_{zk}+\left(\omega_{xk}\omega_{yk}^{2}\omega_{zk}-\omega_{xk}\omega_{yk}{\dot{\omega }}_{xk}\right)\frac{\omega_{xk}\omega_{zk}{\dot{\omega }}_{xk}-\omega_{xk}\omega_{yk}\omega_{zk}^{2}}{{\dot{\omega }}_{xk}{\dot{\omega }}_{yk}+\omega_{xk}\omega_{yk}\omega_{zk}^{2}}};\\ I_{yy}=I_{zz}\frac{\omega_{xk}\omega_{zk}{\dot{\omega }}_{xk}+\omega_{xk}\omega_{yk}\omega_{zk}^{2}}{{\dot{\omega }}_{xk}{\dot{\omega }}_{yk}+\omega_{xk}\omega_{yk}\omega_{zk}^{2}}+\frac{M_{y}{\dot{\omega }}_{xk}-M_{x}\omega_{xk}\omega_{zk}}{{\dot{\omega }}_{xk}{\dot{\omega }}_{yk}+\omega_{xk}\omega_{yk}\omega_{zk}^{2}};\\ I_{xx}=\frac{M_{x}+\omega_{yk}\omega_{zk}I_{yy}-\omega_{yk}\omega_{zk}I_{zz}}{{\dot{\omega }}_{xk}}. \end{array}\right.$$

Now, by estimating the angular velocity and angular acceleration of the space debris object using magnetometer measurements and the moment from controlled action, it is possible to estimate the inertia tensor components from system (7) in the construction coordinate system of the magnetometer.

**Figure 4:**
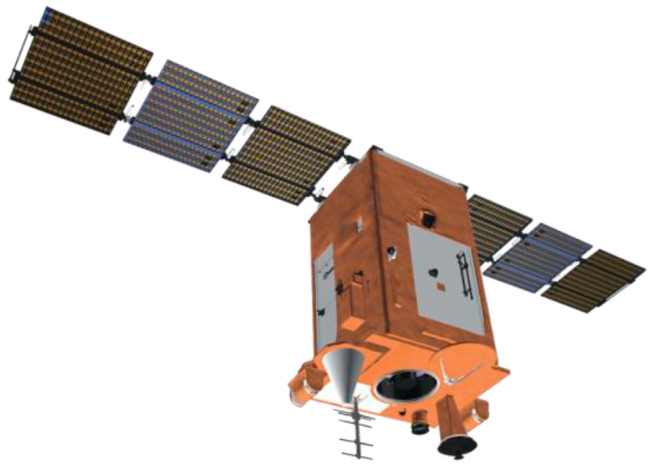
Appearance of the Aist-2D small spacecraft for remote sensing of the Earth [25].

**Figure 5:**
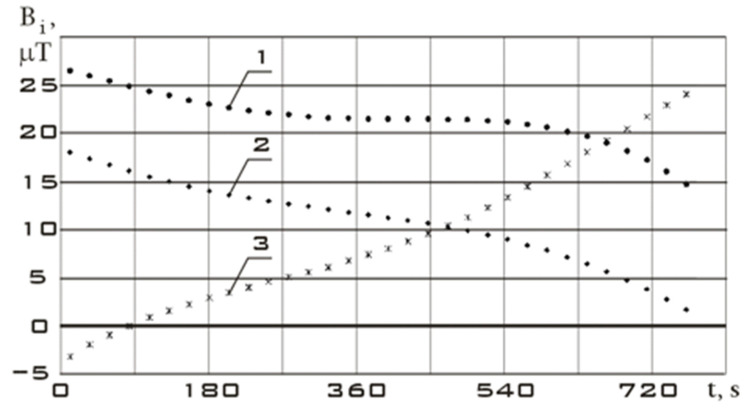
Components of the Earth’s magnetic field induction vector in the magnetometer’s structural coordinate system in stabilization mode: 1 is *B_x_*; 2 is *B_y_*; 3 is *B_z_.*

Let us transform the inertia tensor in accordance with the Huygens–Steiner theorem upon transition to the main body axis system of the space debris object. In the considered case, the axes of the main body axis system of the space debris object and the magnetometer’s structural coordinate system are parallel (Figure 3 and Figure 4). The *y* and *y_b_* axes are offset from each other (Figure 5). Therefore, we have:

(8) $$\hat{I}=\left[\begin{array}{ccc} I_{xx} & 0 & 0\\ 0 & I_{yy}+ma^{2} & 0\\ 0 & 0 & I_{zz}+ma^{2} \end{array}\right],$$

where *m* is the mass of the space debris object; *a* is the distance between the *y* and *y_b_* axes of the main body axis system of the space debris object and the magnetometer’s structural coordinate system (Figure 3).

In this particular case, the components of the inertia tensor are relatively easy to find. Let us illustrate it with an example in the next section of the paper.

## 3. Numerical Simulation for the Aist-2D Small Spacecraft

**Figure 6:**
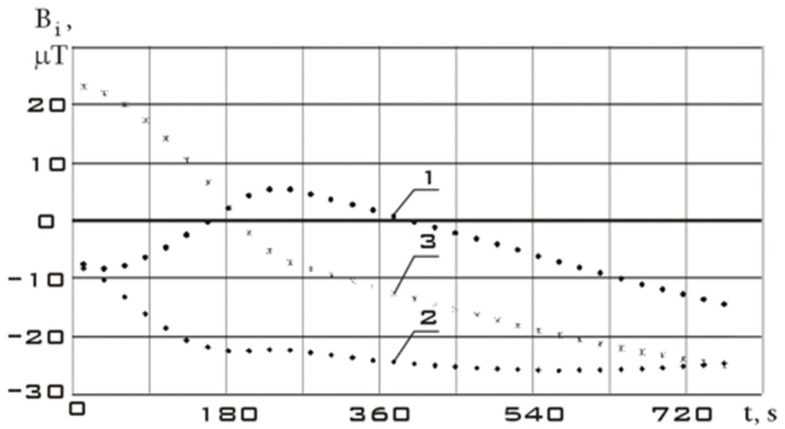
Components of the Earth’s magnetic field induction vector in the magnetometer’s structural coordinate system in reorientation mode: 1 is *B_x_*; 2 is *B_y_*; 3 is *B_z_.*

Let us consider the Aist-2D small spacecraft for remote sensing of the Earth as an example to estimate the inertia tensor components (Figure 6 [25]).

The main parameters of the Aist-2D small spacecraft for remote sensing of the Earth are presented in Table 1 [26].

Modern measuring instruments provide high accuracy in measuring the components of the Earth’s magnetic field induction vector [27]. Therefore, their application can provide an effective estimation of the inertia tensor components for the space debris object. Thus, proton precession magnetometers and optically pumped magnetometers have a sensitivity of about 10–50 pT, an absolute accuracy of about 0.1–1.0 nT, and a dynamic range of 1–100 μT [28].

In this case, the following algorithm is used:

– The fixing of the magnetometers on the space debris object, the construction axes of which are parallel to the axes of the main connected coordinate system of the space debris object;
– The implementation of a controlled impact on the space debris object;
– Carrying out measurements with a uniform step sufficient for the subsequent correct restoration of a continuous signal;
– The restoration of continuous dependences of the Earth’s magnetic field induction vector components via their discrete measurements;
– The estimation of the angular velocity and angular acceleration of the space debris object as a result of a controlled impact;
– The estimation of the space debris object inertia tensor components using dynamic Euler equations.

The Aist-2D small spacecraft is equipped with three-component magnetometers. Their measurements are used as experimental data.

Let us consider the stabilization section of the Aist-2D small spacecraft as the initial section before the controlled action. The measurement data for this section are shown in Figure 5. Time t = 0 corresponds to 31 July 2016, 14:35:46 Moscow time.

Let us choose the section of reorientation of the Aist-2D small spacecraft as a section with controlled action. The measurement data for this section are shown in Figure 6. Time t = 0 corresponds to 31 July 2016, 19:34:28 Moscow time.

To correctly estimate the derivative of the Earth’s magnetic field induction vector components, it is necessary to have continuous dependences of these components on time. These dependencies are then used in Formulas (1) and (2) to determine the vector of angular velocity and rotational acceleration of the space debris object in the magnetometer’s structural coordinate system.

Let us restore discrete measurements to continuous dependencies using the Kotelnikov series [29], since there are measurement data at regular intervals:

(9) $$B_{j}(t)=\sum_{k=-\infty }^{\infty }B_{jk}\frac{\sin\;\left[\frac{\pi }{\Delta t}\left(t-k\;\Delta t\right)\right]}{\frac{\pi }{\Delta t}\left(t-k\;\Delta t\right)},$$

where *j* = *x*, *y*, *z*; *B_jk_* are the measurements at the time *t_k_*; $\Delta t=\Delta t_{k}$ is the uniform step between measurements.

**Figure 7:**
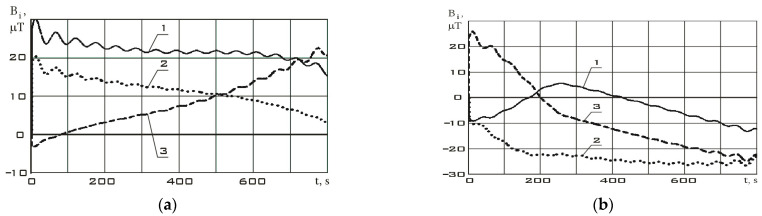
Continuous dependencies of the Earth’s magnetic field induction vector components in the magnetometer’s structural coordinate system, restored using the Kotelnikov series (9): (**a**) in stabilization mode (Figure 5); (**b**) in reorientation mode (Figure 6) 1 is *B_x_*; 2 is *B_y_*; 3 is *B_z_*.

**Figure 8:**
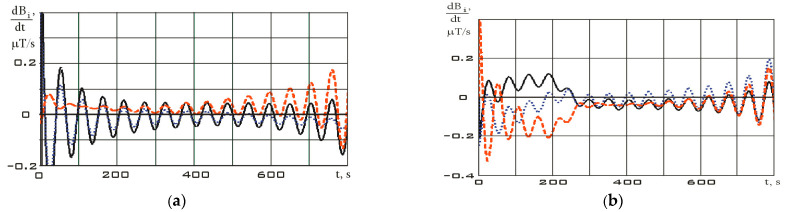
Derivatives of continuous dependencies of the Earth’s magnetic field induction vector components in the magnetometer’s structural coordinate system (Figure 7): (**a**) in stabilization mode; (**b**) in reorientation mode *dB_x_*/*dt* (black); *dB_y_*/*dt* (blue); *dB_z_*/*dt* (red).

Continuous dependencies corresponding to Figure 5 and Figure 6 obtained using the Kotelnikov series (9) are shown in Figure 7. The derivatives of these functions are shown in Figure 8.

The variation ranges of the Earth’s magnetic field induction vector components in the reorientation mode are much wider than in the stabilization mode (Figure 7). It should be noted that the variation ranges of derivatives in different modes are comparable. However, the analysis of Figure 7 shows that in the stabilization mode, the derivatives fluctuate around zero with a sign change. In the reorientation mode, the derivatives have the same sign for a long period of time. This can be explained by the fact that in the stabilization mode, there are random fluctuations in the orientation angles with a change in the sign of the angular velocity. In the reorientation mode, the angular position of the small spacecraft purposefully changes. This is achieved by the fact that the angular velocity has the same sign for a significant period of time. Thus, we can refer to the correct restoration for the continuous dependences of the Earth’s magnetic field induction vector components using the Kotelnikov series (9).

**Figure 9:**
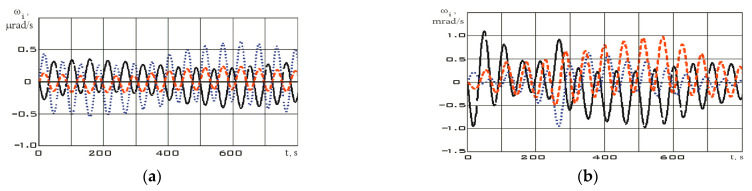
Dependences for the components of the angular velocity vector in the magnetometer’s structural coordinate system, estimated by Equation (2): (**a**) in stabilization mode; (**b**) in reorientation mode *ω_x_* (black); *ω_y_* (blue); *ω_z_* (red).

Let us further estimate the angular velocity by using Formula (2). The estimation results are shown in Figure 9.

**Figure 10:**
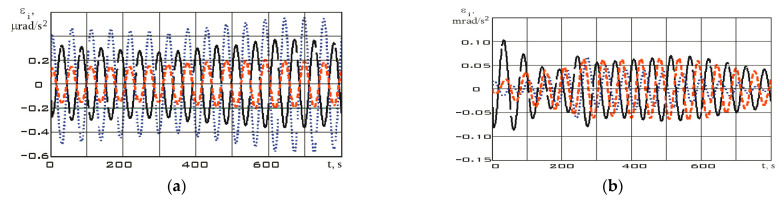
Dependences for the components of the angular acceleration vector in the magnetometer’s structural coordinate system: (**a**) in stabilization mode; (**b**) in reorientation mode *ε_x_* (black); *ε_y_* (blue); *ε_z_* (red).

The derivative of the angular velocity—angular acceleration—will have the form shown in Figure 10.

**Figure 11:**
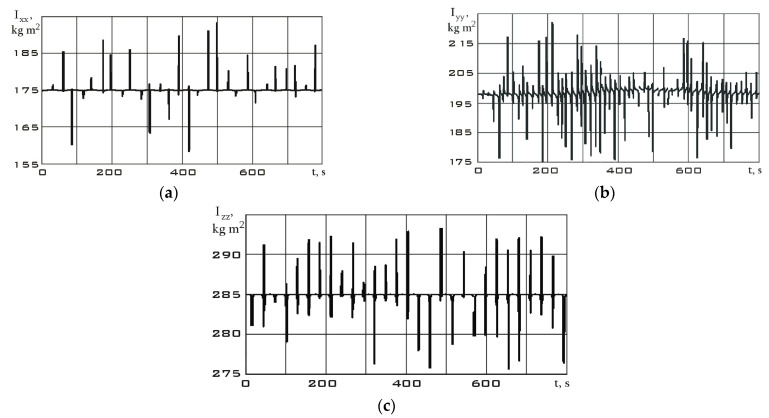
Dependences for the diagonal components of the inertia tensor in the magnetometer’s structural coordinate system in stabilization mode: (**a**) *I_xx_*; (**b**) *I_yy_*; (**c**) *I_zz_*.

**Figure 12:**
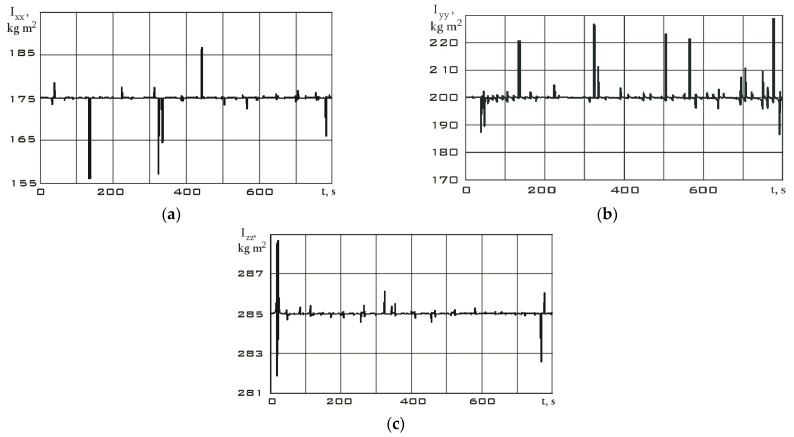
Dependences for the diagonal components of the inertia tensor in the magnetometer’s structural coordinate system in reorientation mode: (**a**) *I_xx_*; (**b**) *I_yy_*; (**c**) *I_zz_*.

The values of the angular velocity and angular acceleration in the stabilization mode are significantly lower than in the reorientation mode (Figure 9). This fact is an important difference between different modes. Let us estimate the dependences for the diagonal components of the Aist-2D small spacecraft inertia tensor by using system (7) according to the measurement data. These dependencies are shown in Figure 11 for the stabilization mode and in Figure 12 for the reorientation mode.

Bursts on the diagonal components of the inertia tensor graphs are associated with both measurement errors and approximation errors of these measurements by the Kotelnikov series (9). Small oscillations in the dependences can be explained by the errors in the attachment of measuring equipment relative to the main body axis system, as well as by natural oscillations of the solar panels of the Aist-2D small spacecraft. These oscillations influenced the components of the inertia tensor and provided them with a dynamic component. In general, upon analyzing Figure 11 and Figure 12, we can state a fine precision of the results with data from Table 1. It can also be seen that in the reorientation mode, the diagonal components of the inertia tensor are estimated more accurately. This is due to the fact that the moment from the executive bodies of the Aist-2D small spacecraft (flywheel engines) was determined more accurately than the moment from many disturbing factors in the stabilization mode. The error of this method in the given numerical example can be estimated correctly only in the case of the inertia tensor components’ constancy over the entire measurement time. Then, the interval estimation for the stabilization and orientation modes, respectively, has the form:

$$\left\{\begin{array}{l} I_{xx}\left(\beta =0,95\right)\in \left[167,\;183\right];\\ I_{yy}\left(\beta =0,95\right)\in \left[190,\;210\right];\\ I_{zz}\left(\beta =0,95\right)\in \left[280,\;290\right]. \end{array}\right.\left\{\begin{array}{l} I_{xx}\left(\beta =0,99\right)\in \left[168,\;182\right];\\ I_{yy}\left(\beta =0,99\right)\in \left[195,\;205\right];\\ I_{zz}\left(\beta =0,99\right)\in \left[282,\;287\right]. \end{array}\right.$$

Here, *β* is the confidence probability.

In fact, due to the natural oscillations of solar panels, the moments of inertia will not remain constant. Error estimation in such a situation is complex and is a random process.

## 4. Conclusions

Thus, as a result of the investigations carried out in the paper, a theoretical estimation of the diagonal components of the space debris object inertia tensor was obtained in the simple case of attaching the measuring equipment on this object. It was assumed that the structural axes of the measuring equipment coincide with the main body axes of the space debris object. As an example, the Aist-2D small spacecraft for the remote sensing of the Earth was taken. Its example demonstrates the possibility of estimating the diagonal components of the inertia tensor using the measurement data of the Earth’s magnetic field induction vector. The average values of the disturbing factors (in the stabilization mode) and the moments of flywheel engines (in the reorientation mode) were chosen as the controlled action on the small spacecraft. The results showing a fine precision with the diagonal components of the inertia tensor of the Aist-2D small spacecraft are obtained. The results of the work can be used in estimating the inertia tensor components of space debris objects. This can be useful when implementing the missions to clean up near-Earth space.

## Figures and Tables

**Table 1:** The main parameters of the simulated Aist-2D spacecraft [26].

Parameter	Designation	Value	Dimension
Mass	*m*	530	kg
Axial moments of inertia	*I_xx_* *I_yy_* *I_zz_*	175 200 285	kg·m^2^
Maximum control torque	*M*	0.2	N·m

## Data Availability

Not applicable.
